# Mechanistic Approach on Melatonin-Induced Hormesis of Photosystem II Function in the Medicinal Plant *Mentha spicata* †

**DOI:** 10.3390/plants12234025

**Published:** 2023-11-29

**Authors:** Michael Moustakas, Ilektra Sperdouli, Ioannis-Dimosthenis S. Adamakis, Begüm Şaş, Sumrunaz İşgören, Julietta Moustaka, Fermín Morales

**Affiliations:** 1 Department of Botany, Aristotle University of Thessaloniki, 54124 Thessaloniki, Greece; begum.sas99@gmail.com (B.Ş.); isgorensn@gmail.com (S.İ.); 2 Institute of Plant Breeding and Genetic Resources, Hellenic Agricultural Organisation-Demeter (ELGO-Demeter), 57001 Thessaloniki, Greece; ilektras@bio.auth.gr; 3 Section of Botany, Department of Biology, National and Kapodistrian University of Athens, 15784 Athens, Greece; iadamaki@biol.uoa.gr; 4 School of Life Sciences, Faculty of Biotechnology, ITMO University, Kronverkskiy Prospekt 49, 19710 Saint-Petersburg, Russia; 5 Department of Molecular Biology and Genetics, Istanbul Kültür University, Ataköy 7-8-9-10, 34158 Bakırköy, Turkey; 6 Department of Food Science, Aarhus University, 8200 Aarhus, Denmark; julietta_moustaka@food.au.dk; 7 Instituto de Agrobiotecnología (IdAB), CSIC-Gobierno de Navarra, Avda. de Pamplona 123, 31192 Mutilva, Navarra, Spain

**Keywords:** chlorophyll content, reactive oxygen species, electron transport rate, non-photochemical quenching, PSII photochemistry, reaction centers, excitation pressure, stomatal closure, excess excitation energy

## Abstract

Melatonin (MT) is considered a new plant hormone having a universal distribution from prokaryotic bacteria to higher plants. It has been characterized as an antistress molecule playing a positive role in the acclimation of plants to stress conditions, but its impact on plants under non-stressed conditions is not well understood. In the current research, we evaluated the impact of MT application (10 and 100 μM) on photosystem II (PSII) function, reactive oxygen species (ROS) generation, and chlorophyll content on mint (*Mentha spicata* L.) plants in order to elucidate the molecular mechanism of MT action on the photosynthetic electron transport process that under non-stressed conditions is still unclear. Seventy-two hours after the foliar spray of mint plants with 100 μM MT, the improved chlorophyll content imported a higher amount of light energy capture, which caused a 6% increase in the quantum yield of PSII photochemistry (Φ*_PSII_*) and electron transport rate (ETR). Nevertheless, the spray with 100 μM MT reduced the efficiency of the oxygen-evolving complex (OEC), causing donor-side photoinhibition, with a simultaneous slight increase in ROS. Even so, the application of 100 μM MT decreased the excess excitation energy at PSII implying superior PSII efficiency. The decreased excitation pressure at PSII, after 100 μM MT foliar spray, suggests that MT induced stomatal closure through ROS production. The response of Φ*_PSII_* to MT spray corresponds to a J-shaped hormetic curve, with Φ*_PSII_* enhancement by 100 μM MT. It is suggested that the hormetic stimulation of PSII functionality was triggered by the non-photochemical quenching (NPQ) mechanism that stimulated ROS production, which enhanced the photosynthetic function. It is concluded that MT molecules can be used under both stress and non-stressed conditions as photosynthetic biostimulants for enhancing crop yields.

## 1. Introduction

Photosynthesis is a fundamental process to plant growth and development, but the plant’s capability to achieve high photosynthetic activity simply depends on the environmental conditions [1]. Enhancing photosynthetic efficiency and improving crop performance stand as crucial and highly significant research challenges [2,3,4]. Improving the quantum yield of photosystem II (PSII) stands as a pathway toward achieving increased efficiency and productivity in photosynthesis [5].

Photosystem II (PSII) uses solar energy to provide electrons by oxidizing water. At PSII in the oxygen-evolving complex (OEC), the oxidation of H_2_O results in oxygen (O_2_), protons (H^+^), and electrons (e^−^) [6]. The e^−^ are transferred to NADP^+^, and coupled with this transfer, the proton gradient that is established drives the synthesis of ATP [6,7]. The activity of PSII is regularly censored by chlorophyll *a* fluorescence measurements [8,9,10,11]. Chlorophyll *a* fluorescence analysis is used extensively for acquiring information regarding the amount of absorbed light energy used for photochemistry (Φ*_PSII_*), the amount of regulated non-photochemical energy loss in PSII (Φ*_NPQ_*), and the amount of nonregulated energy loss in PSII (Φ*_NO_*) [12,13,14]. The sum of Φ*_PSII_* + Φ*_NPQ_* + Φ*_NO_* is equal to 1 [12].

During the conversion of the light energy to chemical energy, reactive oxygen species (ROS), such as hydrogen peroxide (H_2_O_2_), superoxide anion radical (O_2_^•−^), and singlet-excited oxygen (^1^O_2_), are constantly produced [7,15,16,17]. However, they are scavenged by different antioxidant mechanisms [15,16,17,18,19,20]. When ROS production is not well adjusted by the antioxidant mechanisms, photooxidative stress develops [21].

Melatonin (MT) is an indole molecule (*N*-acetyl-5-methoxytryptamine) naturally appearing in roots, leaves, fruits, and seeds [22,23], which was first discovered in the animal kingdom [24]. Melatonin in plants, which is called also phytomelatonin [25], was detected in 1995 by various research groups [22,26,27,28]. The MT molecule plays crucial roles in an extensive variety of physiological processes, e.g., germination, root and shoot growth, photosynthesis, stomatal closure, osmoregulation, secondary metabolism, leaf senescence, circadian cycle regulation, flowering, and fruit setting, and in the protection against biotic and abiotic factors [29,30,31,32,33]. The identification in the model plant *Arabidopsis thaliana* of the first plant melatonin receptor, named PHYTOMELATONIN RECEPTOR 1 (AtPMTR1) [34], unlocked the door to be considered a new plant hormone [29]. Melatonin has been shown to have a universal distribution from prokaryotic bacteria to higher plants, being a phylogenetically conserved molecule [35]. Melatonin activates or deactivates certain metabolic pathways, not merely by regulating gene and protein expression but also through post-translational modifications of proteins [36]. It has been characterized as an antistress molecule playing a positive role in a number of environmental stresses, e.g., in low and high temperatures, salinity, drought, toxic chemicals, UV radiation, fungal diseases, and plant–pathogen interactions [37,38]. Melatonin is related to plant hormones, e.g., abscisic acid (ABA), cytokinins (CTK), gibberellins (GAs), ethylene (ETH), indole acetic acid (IAA), jasmonic acid (JA), brassinosteroids (BR), salicylic acid (SA), and strigolactone (SL) [39,40]. Plants have been found to possess much higher MT levels compared to animals, possibly as a compensatory response to their lack of mobility, to withstand harmful environmental conditions [40]. High MT concentrations have been measured in widespread beverages like tea, coffee, beer, and wine, and also in popular crops like wheat, rice, corn, oats, and barley [40].

Exogenous application of MT can penetrate the plasma membranes increasing the endogenous MT levels [23,41]. Endogenous MT is produced from tryptophan as an intermediate product of the shikimate pathway in the chloroplasts [42]. Melatonin under diverse stress conditions has a fundamental function in preserving the chlorophyll molecules and the photosynthetic function [43]. Additionally, MT interacts with other molecules like ROS, nitric oxide (NO), and Ca^2+^ to regulate the redox network [44,45]. Melatonin and ROS signaling have been shown to be interrelated coordinately [30]. Melatonin-induced plant stress tolerance is linked with up-regulation of stress-induced transcription factors [46]. Melatonin performs a key role in protein quality control in plants and thus functions as a pleiotropic molecule under both non-stress and stress conditions [46].

Melatonin (MT) has been extensively reported to contribute to the acclimation of plants to stress conditions [47]. The positive regulation of MT on photosynthetic efficiency and redox homeostasis under stress conditions has been frequently confirmed [48,49]. Under saline-alkali stress conditions, exogenous MT increased the efficiency of light energy capture and electron transport and improved soybean photosynthesis [50]. In rice plants under salt stress conditions, exogenous MT enhanced photosynthetic function by improving antioxidant capacity, increasing the xanthophyll pool size, and enhancing photosynthetic enzyme activities [47]. Furthermore, exogenous MT application increased strawberry fruit yield and quality under salinity stress [42]. During chilling stress, exogenous MT enhanced violaxanthin de-epoxidase activity accelerating the photoprotective heat dissipation of excitation energy, i.e., the non-photochemical quenching (NPQ), mitigating photoinhibition [51]. Ιn grafted *Carya cathayensis* plants under drought stress, MT regulated metabolic processes, including photosynthesis, antioxidant system, and gene expression [52]. Recently, Karumannil et al. [33] reviewed the molecular mechanisms of MT impact on photosynthetic function in different environmental conditions. However, the molecular mechanisms of the possible interaction between MT and photosynthetic function under non-stressed conditions have seldom been studied [53].

In the current study, we evaluated the consequences of exogenous MT application on the PSII function of *Mentha spicata* plants, under non-stressed conditions. We also evaluated the impact of MT application on ROS generation, and chlorophyll content, in order to elucidate the molecular mechanism of MT action on photosynthetic electron transport that under non-stressed conditions is still unclear.

## 2. Results

### 2.1. Melatonin Impact on Chlorophyll Content

**Figure 1:**
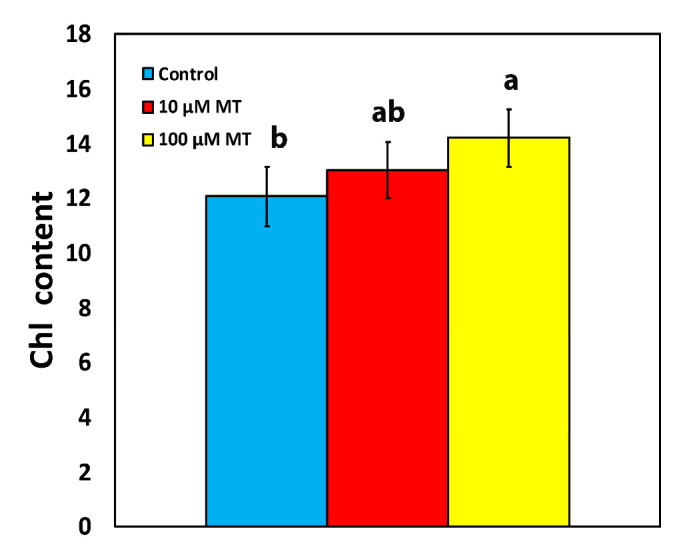
Changes in the chlorophyll content of *Mentha spicata* leaves 72 h after the spray with 10 and 100 μM MT, in comparison to control leaves (sprayed with distilled water). Different lowercase letters symbolize statistical differences (*p* < 0.05). The error bars in columns symbolize SD.

The chlorophyll content of mint plants, 72 h after the spray with 10 μM melatonin (MT) did not differ from those that were sprayed with distilled water (dH_2_O) (Figure 1). However, an 18% increase (*p* < 0.05) in chlorophyll content was observed in plants that were sprayed with 100 μM MT compared to control plants (Figure 1).

### 2.2. Changes in the Efficiency of the Oxygen Evolving Complex and the Maximum Efficiency of PSII Photochemistry by Melatonin

**Figure 2:**
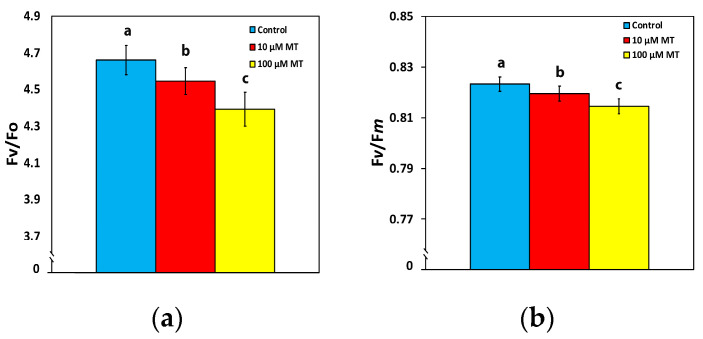
Changes in the efficiency of the oxygen-evolving complex (OEC) (F*v*/F*o*) (**a**), and the maximum efficiency of PSII photochemistry (F*v*/F*m*) (**b**), 72 h after the spray of *Mentha spicata* leaves with 10 and 100 μM MT, in comparison to control leaves (sprayed with distilled water). Different lowercase letters symbolize statistical differences (*p* < 0.05). The error bars in columns symbolize SD.

A malfunction of the oxygen-evolving complex (OEC) was observed in mint plants, 72 h after the spray with MT, showing a decreased efficiency of 2.5% (*p* < 0.05) at 10 μM MT and of 6% (*p* < 0.05) at 100 μM MT, compared to control values (Figure 2a). An analogous pattern was observed in the maximum efficiency of PSII photochemistry (F*v*/F*m*), with a decreased efficiency of 0.5% (*p* < 0.05) at 10 μM MT and of 1% (*p* < 0.05) at 100 μM MT, compared to plants sprayed with dH_2_O (Figure 2b).

### 2.3. Partitioning of the Absorbed Light Energy after Foliar Application of Melatonin

To estimate the partitioning of the captured light energy at PSII, we assessed the effective quantum yield of PSII photochemistry (Φ*_PSII_*), the quantum yield of regulated non-photochemical energy loss in PSII (Φ*_NPQ_*), and the quantum yield of non-regulated energy loss in PSII (Φ*_NO_*), with their sum (Φ*_PSII_* + Φ*_NPQ_* + Φ*_NO_*) to be equal to 1 [12].

**Figure 3:**
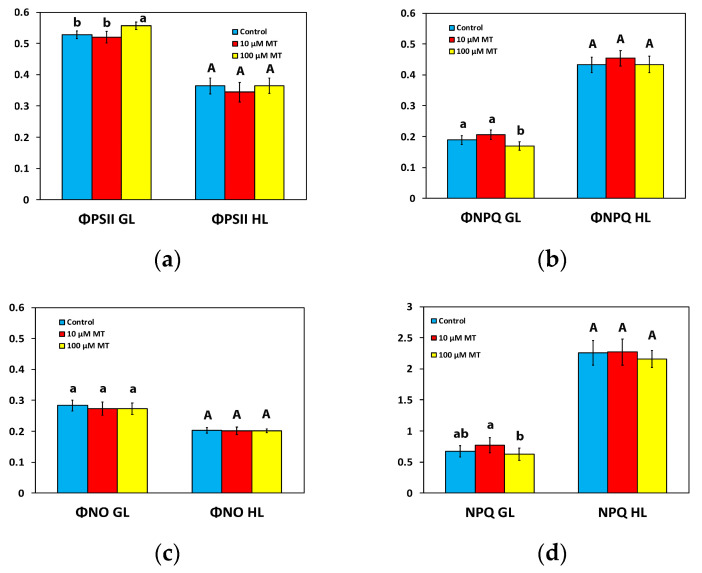
Changes in the allocation of the absorbed light energy; the effective quantum yield of PSII photochemistry (Φ*_PSII_*) (**a**), the quantum yield of regulated non-photochemical energy loss in PSII (Φ*_NPQ_*) (**b**), the quantum yield of non-regulated energy dissipated in PSII (Φ*_NO_*) (**c**); and the photoprotective heat dissipation of excitation energy, i.e., the non-photochemical quenching (NPQ) (**d**); assessed all at the growth light intensity (GL, 200 μmol photons m^−2^ s^−1^), and at a high light intensity (HL, 1000 μmol photons m^−^^2^ s^−^^1^), 72 h after the spray of *Mentha spicata* leaves with 10 and 100 μM MT, compared to control leaves. Different lowercase or uppercase letters symbolize statistical differences (*p* < 0.05). The error bars in columns symbolize SD.

The Φ*_PSII_* of mint plants 72 h after the spray with 10 μM MT did not differ from those that were sprayed with dH_2_O (Figure 3a) at the growth light intensity (GL 200 μmol photons m^−^^2^ s^−^^1^) and at high light intensity (HL, intensity 1000 μmol photons m^−^^2^ s^−^^1^). In contrast, in mint plants, 72 h after the spray with 100 μM MT, Φ*_PSII_* increased (*p* < 0.05) by 6% at the GL intensity, but there was no difference at the HL intensity compared to plants that were sprayed with dH_2_O (Figure 3a).

Φ*_NPQ_*, at both the GL intensity and the HL intensity, of mint plants sprayed with 10 μM MT did not differ from those that were sprayed with dH_2_O (Figure 3b). However, in mint plants, 72 h after the spray with 100 μM MT, Φ*_NPQ_* decreased (*p* < 0.05) by 10% at the GL intensity, but it did not differ from those that were sprayed with dH_2_O at the HL intensity (Figure 3b).

MT treatment had no impact on the quantum yield of non-regulated energy loss in PSII (Φ*_NO_*) at both the GL intensity and the HL intensity (Figure 3c).

### 2.4. Changes in Non-Photochemical Quenching by Melatonin Spray

The non-photochemical quenching (NPQ) of mint plants 72 h after the spray with 10 μM MT did not differ from those that were sprayed with dH_2_O at both the GL and the HL intensity (Figure 3d). In contrast, in mint plants, 72 h after the spray with 100 μM MT, NPQ decreased (*p* < 0.05) by 7% at the GL intensity, but there was no difference at the HL intensity compared to plants that were sprayed with dH_2_O (Figure 3d).

### 2.5. Melatonin Impact on PSII Reaction Centers and Their Efficiency

**Figure 4:**
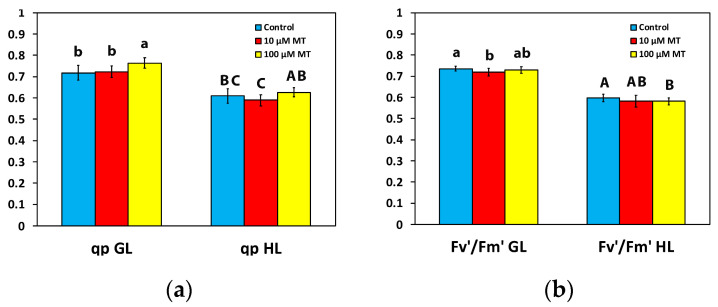
Changes in the fraction of open PSII reaction centers (q*p*), a measure of the redox state of quinone A (Q_A_) (**a**), and the efficiency of excitation energy capture by the open PSII reaction centers (F*v*′/F*m*′) (**b**); assessed all at the growth light intensity (GL, 200 μmol photons m^−2^ s^−1^), and at a high light intensity (HL, 1000 μmol photons m^−^^2^ s^−^^1^), 72 h after the spray of *Mentha spicata* leaves with 10 and 100 μM MT, in comparison to control leaves (sprayed with distilled water). Different lowercase or uppercase letters symbolize statistical differences (*p* < 0.05). The error bars in columns symbolize SD.

Photochemical quenching (q*p*) that represents the fraction of open PSII reaction centers, or in other words the redox state of quinone A (Q_A_), did not differ at both the GL intensity and the HL intensity, in mint plants sprayed with 10 μM MT compared to those that were sprayed with dH_2_O (Figure 4a). However, in mint plants, 72 h after the spray with 100 μM MT, q*p* increased (*p* < 0.05) by 6% at the GL intensity, but there was no difference at the HL intensity compared to plants that were sprayed with dH_2_O (Figure 4a). The efficiency of open reaction centers (F*v*′/F*m*′) in mint plants sprayed with 10 μM MT decreased at the GL intensity compared to those that were sprayed with dH_2_O but remained the same to controls at the HL intensity (Figure 4b). In contrast, in mint plants sprayed with 100 μM MT, F*v*′/F*m*′ remained the same as controls at the GL intensity (Figure 4b) but decreased at the HL intensity compared to plants that were sprayed with dH_2_O (Figure 4b).

### 2.6. Changes in the Electron Transport Rate and the Excess Excitation Energy by Melatonin Spray

**Figure 5:**
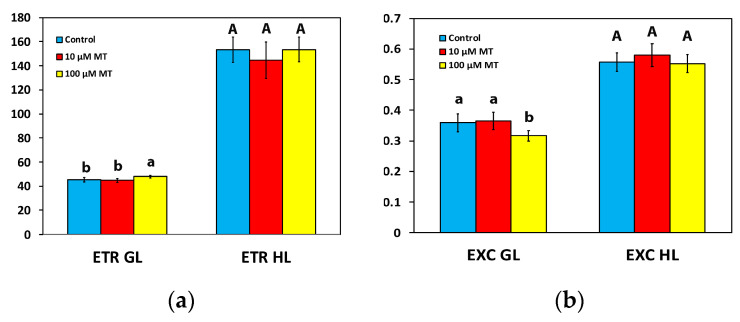
Changes in the electron transport rate (ETR) (**a**), and the relative excess excitation energy at PSII (EXC) (**b**); assessed all at the growth light intensity (GL, 200 μmol photons m^−2^ s^−1^) and a high light intensity (HL, 1000 μmol photons m^−2^ s^−1^), 72 h after the spray of *Mentha spicata* leaves with 10 and 100 μM MT, in comparison to control leaves (sprayed with distilled water). Different lowercase or uppercase letters symbolize statistical differences (*p* < 0.05). The error bars in columns symbolize SD.

The electron transport rate (ETR) of mint plants 72 h after the spray with 10 μM MT did not differ from those that were sprayed with dH_2_O at both the GL intensity and the HL intensity (Figure 5a). In contrast, in mint plants, 72 h after the spray with 100 μM MT, ETR increased (*p* < 0.05) by 6% at the GL intensity, but there was no difference at the HL intensity compared to plants that were sprayed with dH_2_O (Figure 5a).

The excess excitation energy at PSII (EXC) in mint plants, 72 h after the spray with 100 μM MT, decreased (*p* < 0.05) by 12% at the GL intensity, but there was no difference at the HL intensity compared to plants that were sprayed with dH_2_O (Figure 5b). In mint plants sprayed with 10 μM MT, EXC did not differ from those sprayed with dH_2_O at both GL and HL intensity (Figure 5b).

### 2.7. Melatonin Impact on PSII Excitation Pressure

**Figure 6:**
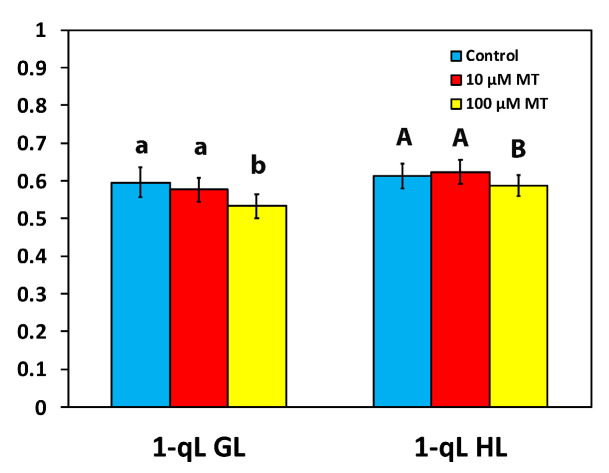
Changes in the excitation pressure at PSII (based on the “lake” model for the photosynthetic unit), assessed at the growth light intensity (GL, 200 μmol photons m^−2^ s^−1^), and at a high light intensity (HL, 1000 μmol photons m^−2^ s^−1^), 72 h after the spray of *Mentha spicata* leaves with 10 and 100 μM MT, in comparison to control leaves (sprayed with distilled water). Different lowercase or uppercase letters symbolize statistical differences (*p* < 0.05). The error bars in columns symbolize SD.

The excitation pressure at PSII, based on the “lake” model for the photosynthetic unit (1-*qL*) in mint plants, 72 h after the spray with 100 μM MT, decreased (*p* < 0.05) by 11% and 4%, at the GL and the HL intensity, respectively, compared to plants that were sprayed with dH_2_O (Figure 6). In mint plants sprayed with 10 μM MT, excitation pressure did not differ from those sprayed with dH_2_O at both GL and HL intensity (Figure 6).

### 2.8. Melatonin Impact on Reactive Oxygen Species Generation

**Figure 7:**
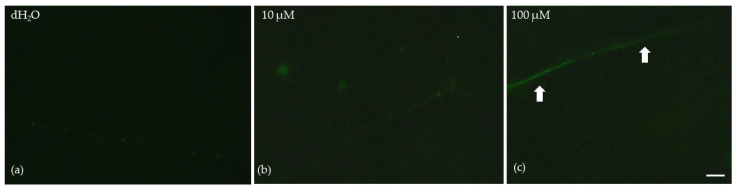
Reactive oxygen species (ROS) production 72 h after the spray of *Mentha spicata* leaves with distilled water (dH_2_O) (**a**), with 10 μM MT (**b**), and 100 μM MT (**c**). The slight light green color denotes a slight ROS generation, arrows point to the midvein. Scale bar: 200 μm.

Low MT foliar spray concentration (10 μM) did not seem to induce any reactive oxygen species (ROS) accumulation (Figure 7b), compared to plants that were sprayed with dH_2_O (Figure 7a). However, foliar spray with 100 μM MT induced a slight increase in ROS generation, especially on the leaf’s midvein (arrows, Figure 7c).

### 2.9. Melatonin-Induced Hormetic Responses of Photosystem II

**Figure 8:**
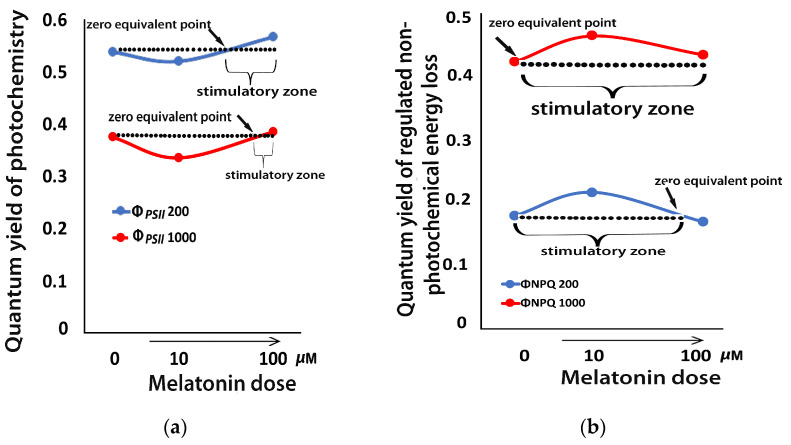
A J-shaped hormetic response curve of Φ*_PSII_* (**a**), and an inverted J-shaped hormetic response curve of Φ*_NPQ_* (**b**), 72 h after the spray of *Mentha spicata* leaves with distilled water (control 0 μM MT) or with 10 and 100 μM MT, assessed either at the growth light intensity (200 μmol photons m^−2^ s^−1^), or at a high light intensity (1000 μmol photons m^−2^ s^−1^).

There was a decline in the effective quantum yield of PSII photochemistry (Φ*_PSII_*) in mint plants, 72 h after the spray with 10 μM MT at both the GL and HL intensity (Figure 8a). This effect changed after the spray with 100 μM MT, with Φ*_PSII_* increasing above the control level at both GL and HL intensity (Figure 8a). This pattern of hormesis corresponds to a J-shaped hormetic response curve (Figure 8a).

In contrast to Φ*_PSII_*, the photoprotective quantum yield of regulated non-photochemical energy loss in PSII (Φ*_NPQ_*), 72 h after the spray with 10 μM MT at both the GL and HL intensity, increased, while it decreased with 100 μM MT (Figure 8b), showing an inverted J-shaped hormetic response pattern (Figure 8b).

## 3. Discussion

Chlorophyll molecules serve as the principal pigments for absorbing light energy and transferring it to the reaction centers (RCs). Melatonin, which, in plants, is synthesized in mitochondria and chloroplasts through two paths that both are based on tryptophan [33], has revealed exceptional protective effects on chlorophyll molecules [53], controlling both the degradation and synthesis of chlorophyll molecules and protecting photosynthetic proteins [53]. A higher chlorophyll content, as we observed after the spray with 100 μM MT (Figure 1), can lead to the formation of larger light-harvesting complexes (LHCs), resulting in an increased capture of light energy and consequently enhancing Φ*_PSII_* and ETR [54,55,56,57,58], as it was detected (Figure 3a and Figure 5a). The observed improvement in photosynthetic function, at the GL following the spray with 100 μM MT, can be attributed to the enhanced light absorption. However, MT spray resulted in the malfunction of the OEC (Figure 2a) that caused donor-side photoinhibition [55,59,60,61], reflected in the reduced F*v*/F*m* (Figure 2b). When the OEC fails to efficiently reduce the chlorophyll molecule at the PSII RC, it results in damaging oxidations in PSII [59]. Consequently, donor-side photoinhibition is often associated with the production of ROS [55,62,63,64]. The minor increase in ROS generation that we observed (Figure 7c), as a result of donor-side photoinhibition (Figure 2b), can be attributed to a malfunction of the OEC (Figure 2a).

The non-photochemical quenching (NPQ) mechanism, by dissipating surplus light energy, serves as a protective measure for the photosynthetic apparatus against the detrimental impacts of ROS [7,56,65]. While a minimal level of ROS is necessary for maintaining life, a slight increase in ROS levels triggers molecular tolerance mechanisms, which are generally considered beneficial. Nevertheless, elevated levels of ROS are recognized as detrimental to plants [7,66,67,68,69,70,71]. NPQ functions as a photoprotective mechanism that inhibits the formation of ROS [72,73,74,75,76]. The reduction of excitation energy dissipation as heat through NPQ by 7%, 72 h after the spray with 100 μM MT (Figure 3d), can explain the slight increase in ROS generation (Figure 7c). However, this slight increase in ROS production can be considered as favorable for triggering defense stress responses [66,77,78]. The surplus light energy dissipated as heat by NPQ reduces the efficiency of PSII photochemistry (down-regulation of PSII) [20,21,74]. The increased excitation energy dissipation as heat through NPQ, 72 h after the spray with 10 μM MT compared to the spray with 100 μM MT (Figure 3d), decreased Φ*_PSII_* (Figure 3a). An increased NPQ, as was observed in mint plants sprayed with 10 μM MT, compared to plants sprayed with 100 μM MT (Figure 3d), decreases the ETR (Figure 5a), preventing the ROS formation (see Figure 7b), which occurs during photoinhibition (Figure 2b) [79].

The increased ETR of mint plants at the GL, following the spray with 100 μM MT, (Figure 5a), could be due to a decreased NPQ (Figure 3d) [79,80]. The observed donor-side photoinhibition, reflected by the reduced F*v*/F*m* (Figure 2b), decreased NPQ (Figure 3d), enhancing the ETR (Figure 5a) [63,81]. The increased effective quantum yield of PSII photochemistry (Φ*_PSII_*), 72 h after the spray with 100 μM MT at the GL intensity (Figure 3a), resulted in increased values of ETR (Figure 5a). Simultaneously, there was a reduction in excess excitation energy at PSII (Figure 5b), indicating enhanced efficiency of PSII. Enhancing photosynthesis is a critical challenge faced by plant scientists, especially in light of the ever-increasing global demand for food [2,82,83]. The ultimate goal of improving photosynthetic efficiency can be accomplished by optimizing the allocation of absorbed light energy [84,85].

As a result of the increased Φ*_PSII_* with 100 μM MT at the GL intensity (Figure 3a), the controlled non-photochemical energy loss in PSII (Φ*_NPQ_*) decreased by 10% (*p* < 0.05) (Figure 3b), while the unregulated energy loss in PSII (Φ*_NO_*) remained unchanged (Figure 3c). An increased Φ*_PSII_* can be attributed either to an increased efficiency of RCs (F*v*′/F*m*′) or/and to an increased number of open RCs (q*_p_*) [86]. The increased Φ*_PSII_*, with 100 μM MT at the GL intensity (Figure 3a), was rather due to the increased fraction of open PSII RCs (q*_p_*) (Figure 4a) than due to increased efficiency of the RCs (F*v*′/F*m*′) (Figure 4b). In *Chara australis* application of 10 μM MT to the artificial pond water, increased Φ*_PSII_* by 34% was attributed to an increased fraction of open PSII RCs, rather than increased efficiency of each RC [87]. More open RCs reflect higher photosynthetic efficiency [87].

The excitation pressure at PSII, based on the “lake” model for the photosynthetic unit (1 − q*_L_*) [12], in mint plants sprayed with 100 μM MT, decreased at both the GL and the HL intensity (Figure 6), which corresponds to diminished stomatal opening [88]. It seems that 100 μM MT could have induced the stomatal closure of mint plants through ROS production [34]. MT-induced stomatal closure is possibly regulated by H_2_O_2_ production and Ca^2+^ influx [34]. Fluctuations in the parameter 1 − q_L_ reflect alterations in the redox state of Q_A_ [12], which act as a signal to the stomatal guard cells [89]. Consistent with this hypothesis, the parameter 1 − q_L_ was linearly correlated to the stomatal conductance in tobacco plants [90]. It seems that stomatal movement is not controlled by the Calvin–Benson cycle but instead by the redox state (Q_A_) [91]. As stomatal closure is a recognized process used by plants to restrict the penetration of pathogens, also known as stomatal immunity [92], MT is now acquiring consideration for its ability to prevent pathogen invasion and induce responses to biotic stress in plants [34,93,94,95].

Hormesis can commonly be exploited as an assessable measure of biological plasticity through adaptive responses under disruption of homeostasis [70,96,97,98]. These adaptive responses, which can be triggered by exposing plants to a low level of a factor that causes disruption of homeostasis, can result in protecting plants through the stimulation of cellular defence mechanisms [66,96,97]. Elucidating the molecular mechanisms that trigger hormesis in plants aims to accomplish higher crop productivity [55,97]. Higher crop productivity can be achieved by more efficient utilization of the absorbed light energy [5,99,100].

Hormetic–biphasic dose–response relationships were commonly observed in plants [55,96,101,102]. Melatonin has been shown to induce biphasic dose–response relationships in a series of studies including plants and animals [102]. In mint plants, MT induced a biphasic dose–response of Φ*_PSII_* with a J-shaped hormetic response curve to be enhanced by 100 μM MT (Figure 8a). Hormetic stimulation of PSII functionality can be triggered by NPQ, which can stimulate ROS production [55,96,103]. The process of NPQ dissipates in a harmless way the excess excitation energy (EXC) and decreases ETR to avoid ROS creation, thus NPQ can control a range of the level of ROS [96,103,104,105]. The slight increase in ROS level, 72 h after the spray with 100 μM MT (Figure 3d), is suggested to trigger the molecular mechanisms that are considered favorable for enhancing photosynthetic function [98,103]. ROS are considered as signaling hormetic molecules, which result in a biphasic dose–response effect on physiological end-points, such as photosynthesis [104,105]. ROS signaling can be favorable and essential for acclimation, regulating different pathways [106,107]. ROS play essential roles in the acclimation process of plants to environmental stress conditions as signal transduction molecules. Hormesis relies highly on the choice of dose range, duration of exposure, and experimental design [55,70,96,103,108,109,110,111,112,113,114]. Consequently, PSII hormetic responses can be observed only in appropriate planned studies [55,96].

Under non-stressed conditions, exogenous MT application in *Chara australis* increased the number of open RCs of PSII, thus improving Φ*_PSII_* [87], as we also observed in *Mentha spicata* plants. In contrast to our results, in which 100 μM MT reduced F*v*/F*m* due to donor-side photoinhibition, Yang et al. [115] suggested that the application of MT might alleviate PSII inhibition and partially display a direct antioxidant effect. They concluded that the application of 200 μM MT in the tea plant (*Camellia sinensis* (L.) Kuntze) stimulated photosynthesis and the expression of genes related to chlorophyll metabolism in a dose-dependent manner [115]. A dose-dependent increase in chlorophyll content was also noticed in our experiments (Figure 1), and enriched chlorophyll content by MT priming under high-temperature stress was observed in the tall fescue [116]. In agreement with our results, MT priming under high-temperature stress increased Φ*_PSII_* by increasing the fraction of RCs and decreased NPQ and the excessive excitation energy [116]. Exogenously applied MT in different crops improved not only crop yield but also quality by active regulation of several traits of plant development and growth, under either stressed or non-stressed conditions [31,53,117,118,119,120,121].

## 4. Materials and Methods

### 4.1. Plant Material, Growth Conditions, and Treatments

Mint (*Mentha spicata* L.) plants were obtained from a plant nursery and transferred to a growth chamber with 16 h light and 8 h dark cycles, 210 ± 10 μmol photons m^−2^ s^−1^ light intensity, 21 ± 1/18 ± 1 °C day/night temperature, and relative humidity 55 ± 5/60 ± 5% day/night.

Melatonin (N-acetyl-5-methoxytryptamine) (MT) was purchased from Sigma-Aldrich (St. Louis, MO, USA) and dissolved in ethanol (20 mg mL^−1^), before being further diluted with ultra-pure water [42,122]. Mint plants were foliar-sprayed until full wetting (15 mL plant^−1^), with 10 μM MT, 100 μM MT, or distilled water (dH_2_O) (control). Control plants were sprayed with dH_2_O with an equal amount of ethanol to that in MT-sprayed plants. To prevent MT from dropping into the soil, the surface of the soil was shielded by an aluminum foil that was detached after the spray. Since MT may be photo-responsive, the plants were sprayed during the dark cycle [123].

Leaf samples from *M. spicata* were taken 72 h after the spray from 4 to 5 plants with 3 independent biological replicates (n = 12–15) for the following measurements.

### 4.2. Chlorophyll Content

Relative chlorophyll content was measured in *Mentha spicata* leaves 72 h after the foliar spray with distilled water (control), 10 μM MT, and 100 μM MT, using a portable Chlorophyll Content Meter (Model Cl-01, Hansatech Instruments Ltd., Norfolk, UK). Values were expressed in relative units [63,124].

### 4.3. Chlorophyll Fluorescence Measurements

Chlorophyll *a* fluorescence was measured in *Mentha spicata* plants using a chlorophyll fluorometer imaging-PAM M-Series (Heinz Walz GmbH, Effeltrich, Germany), as described in detail previously [125]. Fluorescence was excited by blue LED in dark-adapted leaves with saturating pulses (SPs) of 6000 μmol photons m^−2^ s^−1^. Measurements on *M. spicata* leaves were conducted 72 h after the foliar spray with distilled water (control), 10 μM MT, and 100 μM MT. The actinic light (AL) used was 200 μmol photons m^−2^ s^−1^ corresponding to the growth light (GL) or 1000 μmol photons m^−2^ s^−1^ corresponding to a high light (HL) intensity. The chlorophyll fluorescence parameters, described in Table S1, were estimated using Win V2.41a software (Heinz Walz GmbH, Effeltrich, Germany). For each treatment, 12–15 leaves of the same developmental age were measured.

### 4.4. Reactive Oxygen Species Detection

In vivo imaging of ROS in mint leaves was performed 72 h after the foliar spray with distilled water (control), 10 μM MT, and 100 μM MT as described previously [126]. Thirty min after incubation of the leaves in the dark with 25 μM 2′, 7′-dichlorofluorescein diacetate (DCF-DA, Sigma Aldrich, Chemie GmbH, Schnelldorf, Germany), they were observed with a Zeiss AxioImager Z2 epi-fluorescence microscope (Carl Zeiss MicroImaging GmbH, Göttingen, Germany) that was equipped with an AxioCam MRc5 digital camera (Carl Zeiss MicroImaging GmbH, Göttingen, Germany).

### 4.5. Statistical Analysis

Data are presented as mean values ± SD and were tested for normality using the Shapiro–Wilk test and for homogeneity of variance using Levene’s test. The population of variances was not equal, so significant differences between the three treatments were determined using Welch ANOVA followed by a post hoc analysis with the Games–Howell test. All analyses were performed using SPSS version 28.0 (IBM, Chicago, IL, USA) for Windows. Values were considered significantly different at *p* < 0.05.

## 5. Conclusions

We observed a hormetic response of Φ*_PSII_*, which was probably triggered by NPQ that stimulated ROS production at 100 μM MT. The application of 100 μM MT in mint plants increased the chlorophyll content, possibly resulting in increased LHCs and increased light energy capture that enhanced ETR. In addition, 100 μM MT decreased the excess excitation energy at PSII and the excitation pressure at PSII, indicating an improved PSII efficiency. Improving photosynthetic function is of great importance for improving plant productivity and grain yield. Therefore, MT can potentially be used as a photosynthetic biostimulant that can be applied to plants exogenously to enhance crop yields while reducing the use of chemical fertilizers, also under non-stressed conditions.

## Data Availability

The data presented in this study are available in this article.

## Supplementary Materials

The following supporting information can be downloaded at: https://www.mdpi.com/article/10.3390/plants12234025/s1, Table S1: Definitions of the chlorophyll fluorescence parameters used in the experiments.
