# Supplementary material for: Mechanistic Approach on Melatonin-Induced Hormesis of Photosystem II Function in the Medicinal Plant Mentha spicata
Source: Plants (Basel). 2023 Nov 29;12(23):4025. doi: 10.3390/plants12234025 (PMC10708495; doi:10.3390/plants12234025)
Supplement: Supplementary file 1 [file plants-12-04025-s001.zip › plants-2739375-supplementary.pdf]

Mechanistic Approach on Melatonin Induced Hormesis of Photosystem II Function in the Medicinal Plant *Mentha spicata*

Michael Moustakas, Ilektra Sperdouli, Ioannis-Dimosthenis S. Adamakis, Begüm Şaş, Sumrunaz İşgören, Julietta Moustaka and Fermín Morales

Table S1. Definitions of the chlorophyll fluorescence parameters used in the experiments

Parameter	Definition	Calculation
F_v/F_m	Maximum efficiency of PSII photochemistry	$(F_m - F_o)/F_m$
Φ_{PSII}	Effective quantum yield of PSII photochemistry	$(F_m' - F_s)/F_m'$
Φ_{NPQ}	Quantum yield of regulated non-photochemical energy loss in PSII	$F_s/F_m' - F_s/F_m$
Φ_{NO}	Quantum yield of nonregulated energy loss in PSII	F_s/F_m
F_v'/F_m'	Efficiency of open PSII reaction centers	$(F_m' - F_o')/F_m'$
F_v/F_o	Efficiency of the oxygen evolving complex (OEC) on the donor side of PSII	$(F_m - F_o)/F_o$
ETR	Electron transport rate	Φ_{PSII} × PAR × c × abs, where PAR is the photosynthetically active radiation, c is 0.5, and abs is the total light absorption of the leaf taken as 0.84
q_p	Photochemical quenching, representing the redox state of quinone A (Q_A), or in other words the fraction of PSII reaction centers in open state based on the "puddle" model for the photosynthetic unit	$(F_m' - F_s)/(F_m' - F_o')$
NPQ	Non-photochemical quenching reflecting the dissipation of excitation energy as heat	$(F_m - F_m')/F_m'$
EXC	Excess excitation energy	$(F_v/F_m - \Phi_{PSII})/F_v/F_m$
1-qL	The fraction of closed PSII reaction centres based on a lake model for the photosynthetic unit	$1- (q_p \times F_o'/F_s)$
